# The adaptive nature of the plant circadian clock in natural environments

**DOI:** 10.1093/plphys/kiac337

**Published:** 2022-07-27

**Authors:** Madeline W Oravec, Kathleen Greenham

**Affiliations:** Department of Plant and Microbial Biology, University of Minnesota, St. Paul, Minnesota 55108, USA; Department of Plant and Microbial Biology, University of Minnesota, St. Paul, Minnesota 55108, USA

## Abstract

The plant circadian clock coordinates developmental, physiological, and metabolic processes with diel changes in light and temperature throughout the year. The balance between the persistence and plasticity of the clock in response to predictable and unpredictable environmental changes may be key to the clock’s adaptive nature across temporal and spatial scales. Studies under controlled conditions have uncovered critical signaling pathways involved in light and temperature perception by the clock; however, they don’t account for the natural lag of temperature behind photoperiod. Studies in natural environments provide key insights into the clock’s adaptive advantage under more complex natural settings. Here, we discuss the role of the circadian clock in light and temperature perception and signaling, how the clock integrates these signals for a coordinated and adaptive response, and the adaptive advantage conferred by the clock across time and space in natural environments.

## Teaser

The circadian clock orchestrates the precise coordination of plant processes across daily and annual cycles of light and temperature, resulting in adaptation to local environments.

## Introduction

**Figure 1:**
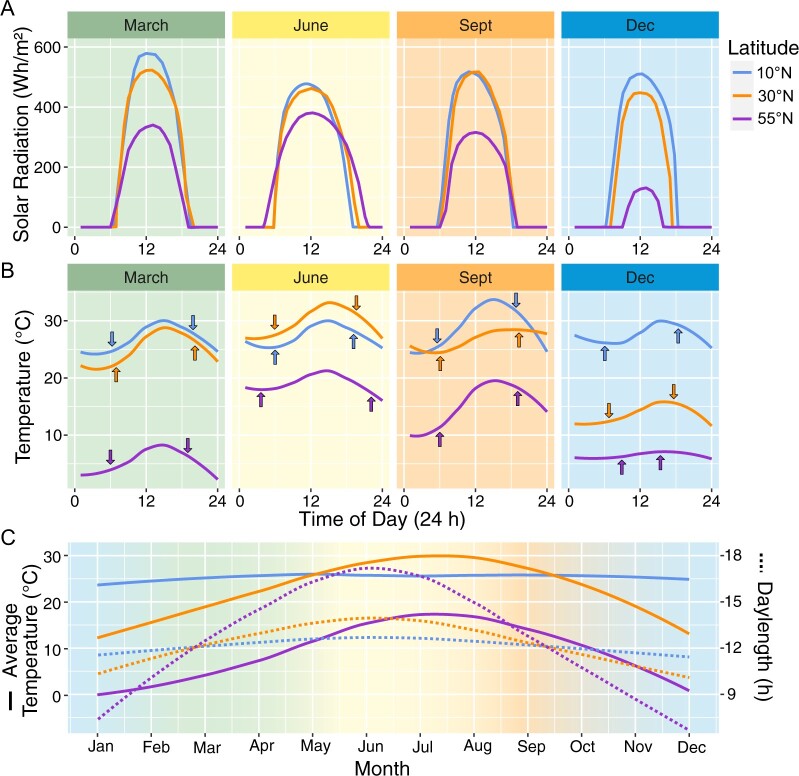
Daily and annual patterns of light and temperature. Sunlight and temperature patterns in locations representing different latitudinal zones, 10°N (Caracas, Venezuela; 10.5°N, 66.9°W), 30°N (New Orleans, LA, USA; 30.0°N, 90.0°W), and 55°N (Copenhagen, Denmark; 55.7°N, 12.6°E). A, Hourly solar radiation (Wh m^−2^) and (B) hourly temperature (°C) for a clear day in each location near the spring equinox (March), summer solstice (June), fall equinox (September), and winter solstice (December) in 2020. Arrows in (B) indicate dawn and dusk. C, Average monthly temperature (°C) and daylength (h) within each location in 2020. Average solar radiation and daylength plots modeled based on data from the “Global Solar Atlas 2.0” (https://globalsolaratlas.info) and Weather Spark (https://weatherspark.com/); temperature data obtained from Time and Date AS (https://www.timeanddate.com/).

Rhythms are innate to our planet, from the earth’s daily rotation on its axis to its yearly orbit around the sun. Temporal and spatial gradients across regional and global landscapes create variation in daily and seasonal cycling of light and temperature (Figure 1). Organisms across all domains and kingdoms have evolved to capture and exploit these patterns through use of an internal circadian oscillator (reviewed in Young and Kay, 2001 and Saini et al. 2019).

For centuries, botanists have been attuned to the daily and seasonal patterns of plant processes, such as floral opening, inspiring Linnaeus’s floral clock (Horologium Florae). Linnaeus proposed a timekeeping garden with 15-min resolution based on the timing of floral opening and closing in different plants (von Linné, 1751). While variation across latitudes and seasons makes this garden concept much more complicated in practice than theory, this timekeeping ability of plants is important for their adaptation to the local environment across days, seasons, years, and geographic ranges.

The plant circadian clock is entrained by exogenous cues from the environment and maintained by coordinated transcriptional–translational feedback loops (Box 1). The circadian oscillator is entrained to light and dark (photocycling) and temperature (thermocycling) cycles and can vary by period (cycle length), phase (timing of peak), and amplitude (half the difference between peak and trough; reviewed in Webb et al., 2019). It is important to distinguish diel versus circadian regulation. Diel regulation signifies responses directly related to daily cues such as photo- or thermo-cycling, while circadian regulation denotes cycling that is maintained under free-running conditions, without the presence of exogenous cues.

Box 1 The plant circadian clock transcriptional network.

The plant circadian clock consists of a series of transcriptional–translational feedback loops that drive rhythmic patterns of activity throughout the day. A tremendous body of work in *A. thaliana* has uncovered the components necessary for oscillator function and we encourage readers to read recent reviews detailing these studies (McClung, 2019; Webb et al., 2019; Nakamichi, 2020; Nohales, 2021). Briefly, the dawn expressed CIRCADIAN CLOCK ASSOCIATED 1 (CCA1) and LATE ELONGATED HYPOCOTYL (LHY) myb-like transcription factors repress the *PSEUDO-RESPONSE REGULATOR* (*PRR)* genes, *GIGANTEA* (*GI)*, and the evening complex (EC) components, *LUX ARRHYTHMO* (*LUX*)*, EARLY FLOWERING 3* (*ELF3*), and *ELF4*, in addition to their own transcription*.* The *PRRs* are sequentially expressed throughout the day starting with *PRR9*, and followed by *PRR7*, *PRR5*, and *PRR1* (also known as *TIMING OF CAB EXPRESSION1*, *TOC1*; Matsushika et al., 2000). The expression of *TOC1* and *PRR5* is promoted by the *NIGHT LIGHT-INDUCIBLE AND CLOCK-REGULATED GENES 1 (LNK1)* and *LNK2* transcriptional co-activators complexed with members of the DNA-binding *REVEILLE* family (*RVE4*, *RVE6*, and *RVE8*; Rawat et al., 2011; Rugnone et al., 2013; Xie et al., 2014; Pérez-García et al., 2015). The LNK–RVE complex(es) also activates the expression of *GI* and the EC. *CCA1* expression is regulated by LIGHT-REGULATED WD1 (LWD1) and LWD2 along with TEOSINTE BRANCHED 1-CYCLOIDEA-PCF20 (TCP20) and TCP22 (Wu et al., 2016). As the *PRRs* are expressed, they repress *CCA1*, *LHY*, and their own transcription. TOC1 represses members of the EC and *GI*, while the EC in turn represses *PRR9*, *PRR7*, and *LUX* (McClung, 2019; Webb et al., 2019; Nakamichi, 2020; Nohales, 2021).

These transcriptional and translational cycles time metabolic processes throughout the day to ensure the coordination of physiology with the external environment (reviewed in Greenham and McClung, 2015). A sunflower (*Helianthus annuus*) turning to the east to prepare to capture light for photosynthesis prior to dawn (Atamian et al., 2016), the calculated degradation of starch during the night in Arabidopsis (*Arabidopsis thaliana*; Graf et al., 2010), and response to abiotic stresses (reviewed in Seo and Mas, 2015) are a few examples of the daily processes controlled by the clock. Changes in daylength (photoperiod) throughout the year impose additional environmental signals that the clock relays to the plant to control seed dormancy (Penfield and Hall, 2009), growth (Ramos-Sánchez et al., 2019), flowering time (Anwer et al., 2020), and senescence (Kim et al., 2018). While the clock dynamically adjusts based on external inputs of light, temperature, and other factors (plasticity), it also maintains accurate timekeeping despite varying environmental conditions (persistence; Matsuzaki et al., 2015). The balance between plasticity and persistence of the clock is likely key to the adaptive advantage conferred by the clock.

Experimentally coupling circadian function with fitness and thus adaptation is a complicated endeavor, given the narrow definitions of both fitness and adaptation (reviewed in Johnson, 2005). Some studies have reported increased fitness associated with a circadian period that matches the environmental rhythm, providing evidence for the adaptive nature of the clock (Ouyang et al., 1998; Yerushalmi et al., 2011); however, most studies address adaptation by proxy, through enhanced stress response, photosynthesis, survival, or rates of growth and development. In this review, we discuss this implied adaptation, addressing the adaptive nature of the plasticity of the clock in response to light and temperature signals and the clock’s role in facilitating plants’ responses to their dynamic environment across spatial and temporal scales.

## Role of the clock in light and photoperiod perception and signaling

Light is one of the most important entraining cues for the clock. Light is an intricate environmental signal that varies in solar radiation intensity, daylength, and quality, and changes depending on latitude, time of year, and time of day (Figure 1). Daylength (photoperiod) predictably changes across the cycle of a year within a latitudinal zone. Solar angle changes across individual days and over the cycle of a year, influencing both light intensity and quality across latitudinal zones (Kotilainen et al., 2020). Additional daily and seasonal variation in light intensity and quality occurs from variable environmental factors, like cloud cover, ozone layer, or canopy structure and cover (Chiang et al., 2019; Kotilainen et al., 2020). The combined signaling of light intensity, quality, and photoperiod across days and seasons must therefore be integrated into the entrainment of the clock.

Light is perceived by five plant light receptors with specific wavelength absorptions: the cryptochromes (CRY1, CRY2), the phototropins (PHOT1, PHOT2), the ZEITLUPE/FLAVIN-BINDING, KELCH REPEAT, F BOX 1/LOV KELCH PROTEIN2 (ZTL, FKF1, LKP2) blue light sensing family, the ultraviolet (UV)-B photoreceptor UV RESISTANCE LOCUS 8 (UVR8), and the red light (R) and far-red light (FR) perceiving phytochromes (PHYA-E; Ahmad and Cashmore, 1993; Somers et al., 1998a; Lin and Todo, 2005; Zoltowski and Imaizumi, 2014; Casal and Balasubramanian, 2019; Legris et al., 2019). These light receptors are responsible for irradiance and spectrum-based signaling to the clock. The clock in turn modulates light responsiveness throughout the day. For example, the clock mediates the light induced responsiveness of the *CHLOROPHYLL A/B-BINDING PROTEIN (CAB)* gene to attenuate light responsiveness overnight and tune responsiveness to dawn, which is dependent on *EARLY FLOWERING 3 (ELF3*; McWatters et al., 2000). This regulation also varies with photoperiod where short-day (SD) conditions elicit a more rapid light induced response (Millar and Kay, 1996). This would suggest an adaptive advantage for being able to maximize photosynthesis when days are short.

**Figure 2:**
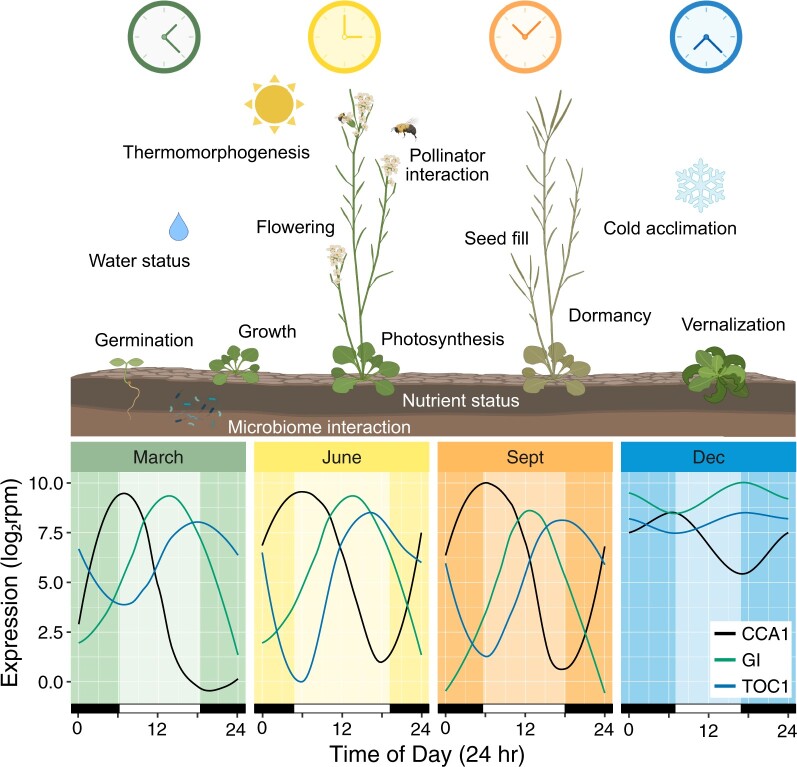
Seasonal clock variation and corresponding physiological influences. Variation in daily expression patterns of core clock genes (*CCA1*, *GI*, *TOC1*) across seasons and various physiological processes influenced by the plant circadian clock across a year. Gene expression patterns adapted from Nagano et al. (2019) from transcriptomic data for natural population of *Arabidopsis halleri* subsp. *gemmifera* in Hyogo, Japan (35.1°N, 134.9°E) on spring equinox (March; high 16°C, low 10°C), summer solstice (June; high 26°C, low 22°C), fall equinox (Sept; high 31°C, low 24°C), and winter solstice (Dec; high 10°C, low 3°C), in 2013. Graphics created with BioRender (https://biorender.com/).

The morning-expressed core clock component *CIRCADIAN CLOCK ASSOCIATED 1* (*CCA1*) is highly sensitive to light. A 1-min pulse of white light given to dark grown Arabidopsis induces the expression of *CCA1* (Liu et al., 2020). This induction is lost in the phytochrome mutants *phyAphyB* and *phyABDE* and the phytochrome light signaling mutant of *FAR-RED-ELONGATED HYPOCOTYL 3 (FHY3)*. FHY3 accumulates within 1 min of light and binds to the promoter to activate *CCA1* expression. This activation is blocked by TIMING OF CAB EXPRESSION 1 (TOC1) binding to FHY3 during the dark period (Liu et al., 2020). The punctual morning expression of *CCA1* is apparent for *Arabidopsis halleri* subsp. *gemmifera* growing in its natural environment across seasons (Nagano et al., 2019; Figure 2). In addition to *CCA1*, clock genes *PSEUDO-RESPONSE REGULATOR 7 (PRR7)*, *GIGANTEA (GI)*, and *NIGHT LIGHT-INDUCIBLE AND CLOCK-REGULATED GENES 1–4 (LNK)* showed the largest induction in response to a light pulse when given at night (Rugnone et al., 2013). This is consistent with the enhanced sensitivity of clock entrainment to nighttime light (Covington et al., 2001) and likely important for sensing seasonal changes in daylength.

Precise sensitivity to dawn means that plants across a landscape may entrain differently depending on their location, size, or proximity to other plants. Expression of morning-expressed *LATE ELONGATED HYPOCOTYL (LHY)* peaked 2 h earlier in 4-month versus 9-month-old sugarcane (*Saccharum* hybrid) along with half of rhythmic expressed transcripts and a 6 h earlier peak in sucrose (Dantas et al., 2021). Dantas et al. (2021) hypothesized that this was due to self-shading in older stands. This hypothesis was consistent with their finding that *LHY* peaked ∼1 h later in plants on west versus east margins of the field and in plants behind versus in front of an artificial wall, corresponding to the delay in full sunlight illumination of ∼1 h and an even greater delay in temperature change (Dantas et al., 2021). These findings suggest that plants across natural landscapes experience different microclimates that can alter photoperiod sensing by the clock. Likewise, different parts of the same plant experience variation in light timing and quality as well as temperature patterns due to self- or canopy-shading. This poses an interesting question of how the clock differentially influences plant performance between canopy and understory plants.

The transition from vegetative growth to flowering has precise seasonal timing to appropriately coordinate flowering with conducive environmental conditions after sufficient growth and resource accumulation for seed production. This coordination requires plants to have a sense of the time of year, which is primarily accomplished through photoperiodic sensing. The molecular mechanisms of flowering have been extensively studied and reviewed (Cao et al., 2021; Freytes et al., 2021). Briefly, in Arabidopsis, photoperiodic flowering is induced by *FLOWERING LOCUS T (FT)* after its transcriptional activation by CONSTANS (CO) and putative orthologs to these genes and pathways have been identified across species (Kardailsky et al., 1999; Kobayashi et al., 1999; Samach et al., 2000; Fan et al., 2014). Light signaling pathways and circadian components control the transcriptional and posttranscriptional regulation of CO resulting in complex photoperiodic flowering control (Suárez-López et al., 2001; Valverde et al., 2004). Induction of flowering is determined based on both external and internal coincidence models, such that external environmental cues align with internal cycles of gene expression. Under SD, *FKF1* and *GI* gene expression peak in the dark with minimal overlap. The FKF1–GI protein complex formation accumulates in light under long-day (LD) conditions when their expression phases overlap (Sawa et al., 2007). The blue light stabilized FKF1–GI complex targets the CYCLING DOF FACTOR (CDF) transcriptional repressors of *CO* for degradation, allowing CO to induce flowering (Sawa et al., 2007; Fornara et al., 2009).

GI is a primary circadian mediator of *CO* expression, and along with ELF3, is essential to photoperiodic flowering in Arabidopsis (Mizoguchi et al., 2005; Anwer et al., 2020). The involvement of GI in flowering induction is conserved across diverse species of angiosperms (reviewed in Mishra and Panigrahi, 2015). Mutations in both *ELF3* and *GI* lead to the loss of photoperiod responsive flowering and reduced sensitivity to photoperiod-dependent hypocotyl growth. In addition, the characteristic expression patterns of *CCA1*, *PRR9*, and *TOC1* under diel conditions are lost in the *elf3-4 gi-158* mutant, consistent with a loss of clock sensing of photoperiod (Anwer et al., 2020).

Putative orthologs of Arabidopsis flowering genes *CO* and *FT* in poplar trees (*Populus* spp.) contribute to the regulation of seasonal bud set and growth cessation (Böhlenius et al., 2006). In poplar, daylength influences *LHY2* expression, accumulating during the night in SD to suppress *FT2* expression and shoot apical growth (Ramos-Sánchez et al., 2019). Two *GI-like* putative paralogs play a key role in maintaining vegetative growth and preventing growth cessation and bud set in LD in poplar, such that knockdown stopped growth and led to premature bud set under LD, while overexpression delayed bud set even under SD conditions (Ding et al., 2018). Delayed bud set is also observed in *lhy* (*lhy-3*; *lhy-8*; *lhy-10*) and *toc1* (*toc1-1*; *toc1-4*; *toc1-5*) mutants with shortened circadian periods (Ibanez et al., 2010). LHY also functions in promoting bud burst in spring, indicated by delayed bud break in *lhy* (*lhy-3*; *lhy-10*) mutants (Ibanez et al., 2010).

Depending on the species and adaptive timing for flowering, other clock genes have variably evolved in photoperiodic responses to induce or repress flowering. For example, the *PRR*s differentially influence photoperiodic flowering of LD and SD flowering plants. While overexpression of PRR5 causes early flowering in LD Arabidopsis (Nakamichi et al., 2007), overexpression of AtPRR5 in SD rice (*Oryza sativa*) delays flowering (Nakamichi et al., 2020). Four *LHY-CCA1-LIKE (LCL)* orthologs were reported in soybean (*Glycine max*), with circadian rhythmicity entrained by both light and temperature patterns (Wang et al., 2020). While both the Arabidopsis *lhy* (*lhy-11*; *lhy-12*; *lhy-13*) *cca1-1* mutants and the quadruple *LCL* loss-of-function mutant in soybean display shortened circadian period, flowering is induced in the Arabidopsis mutants and delayed in the soybean mutant under SD (Mizoguchi et al., 2002; Wang et al., 2020). Natural variation in the *ELF3* homolog (*Heading date 17*, *Hd17*) in rice leads to altered photoperiodic flowering (Matsubara et al., 2012) and *HvELF3* is required to initiate photoperiod-sensitive entrainment in barley (*Hordeum vulgare*; Deng et al., 2015). In soybean, a modern haplotype of *GmPPR3b* inhibits *GmCCA1*, causing the loss of *GmELF3* activation and delayed flowering (Li et al., 2020). These findings are consistent with the role of ELF3 in photoperiodic sensing in Arabidopsis (Anwer et al., 2020). Taken together, it is apparent that clock variation has differing effects across LD and SD plants, which could indicate divergent regulation of flowering downstream of the clock.

## Role of the clock in temperature perception and signaling

The relationship between temperature and the circadian clock is complex and seemingly paradoxical. A property of all oscillators is temperature compensation where the circadian period is maintained despite changes in ambient temperature, thus accommodating variation in weather. Yet, circadian clocks can be entrained by temperature cycles as small as 4°C (Somers et al., 1998b), a competing action to temperature compensation. How a clock can be compensated to and reset by the same temperatures is especially relevant when considering plant responses to temperature fluctuations in the natural environment. While the exact mechanism of temperature compensation isn’t clear, several clock genes including *CCA1, LHY, GI, PRR7*, and *PRR9* are known to be involved (Gould et al., 2006; Salome et al., 2010). Likewise, clock genes *ELF3*, *PRR7*, and *PRR9* are essential for thermocycle entrainment (Salomé and McClung, 2005; Thines and Harmon, 2010).

While ELF3 is a core member of the clock evening complex (EC), its role in thermocycle entrainment is independent of other *EC* genes, *ELF4* and *LUX ARRHYTHMO (LUX)*, indicating its distinct role as a thermosensor within the clock (Nusinow et al., 2011; Zhu et al., 2022). The temperature sensing function of ELF3 is dependent on a variable length polyQ repeat region located within a prion-like domain (Jung et al., 2020). This domain is responsible for the temperature-dependent phase separation of ELF3, leading to inactivation at high temperatures. Temperate grown potato (*Solanum tuberosum*) contains a smaller domain compared to Arabidopsis, and warm adapted *Brachypodium distachyon* lacks it completely. A chimeric Arabidopsis ELF3 with the corresponding sequence of the domain from *B. distachyon* suppresses thermoresponsive flowering (Jung et al., 2020). ELF3 stability is further regulated through B-BOX 18 (BBX18)-dependent targeted degradation at warm temperatures by the E3-ligases XB3 ORTHOLOG 1 IN ARABIDOPSIS THALIANA (XBAT31) and XBAT35 (Zhang et al., 2021a, 2021b). These findings support a key role for ELF3 in adaptive variation in temperature signaling and thermocycle entrainment.

Phytochromes likely also play a role in integrating temperature into the clock. Phytochromes exist in two conformations. The active form (Pfr) is induced by R and is inactivated (to Pr) by both FR and temperature-dependent thermal reversion (reviewed in Klose et al., 2020). Critical to the PHYB Pfr form function is the light-induced formation of photobodies, which is stabilized by the EC-associated PHOTOPERIODIC CONTROL OF HYPOCOTYL 1 (PCH1) providing prolonged growth repression under long nights (Huang et al., 2016a, 2016b). Thermal reversion activity provides a unique temperature sensing property of PHYB that is independent of light and modulated by PCH1 (Jung et al., 2016; Legris et al., 2016; Huang et al., 2019). The thermomorphogenic response is not completely lost in the *phyB* mutant but is in the quintuple *phyABCDE* mutant, suggesting that other phytochromes are also important for temperature sensing (Jung et al., 2016). Modeling experiments predict that PHYB activity is influenced more by temperature when light levels are low (i.e. early morning, shaded, or cloudy conditions) compared to in full sunlight when light is the predominant signal (Sellaro et al., 2019). PHYB has been shown to interact with several clock proteins including ELF3, GI, TOC1, CCA1, LHY, and LUX, and some of these interactions depend on light quality (Yeom et al., 2014). The interaction with LUX occurred under R but not FR, CCA1, and TOC1 preferentially interacted with PHYB under FR, and LHY, GI, and ELF3 interacted with PHYB under both R and FR. The physiological importance of these interactions is not fully known, but they do offer a mechanism for signaling changes in R/FR ratios to the clock (Yeom et al., 2014). Increased cloud cover has been observed in much of the world with the changing climate (Cox et al., 2020), indicating that clock interactions with PHYB may be ever more important, and warranting further studies on the interaction of the clock with phytochromes across dynamic environments.

Much of the work on temperature sensing in plants has focused on either stress conditions or prolonged growth at changes in constant ambient temperature. Circadian studies on natural accessions of Arabidopsis grown at temperatures that span the ambient range (12°C–27°C; Wigge, 2013) have revealed diverse temperature compensation responses across accessions. A general trend found across studies and species is a shortening of period at elevated temperatures (Edwards et al., 2005; Kusakina et al., 2014; Bdolach et al., 2019). A unique *GI* allele in Arabidopsis is associated with more extreme period shortening at high temperature, which is counterbalanced by a unique *ZTL* allele with high-temperature-dependent period lengthening (Kim et al., 2020). The interaction of these unique alleles is reduced at high temperature and, since GI plays a role in ZTL stabilization, this results in altered ZTL accumulation, which is associated with more robust temperature compensation. This suggests that the interaction of GI and ZTL plays a key role in temperature compensation mechanisms (Kim et al., 2020). While these experiments do inform our understanding of temperature compensation, they do not address circadian performance in response to changes in thermocycles, a condition that is more reflective of the natural environment.

Mathematical modeling of clock entrainment and temperature compensation revealed more robust temperature compensation, meaning more stable clock function, in the presence of both temperature and light cycling conditions (Avello et al., 2019). The models indicate that temperature-dependent degradation rates of mRNA and protein across the system play a role in mediating the paradoxical compensation and entrainment properties of the clock. Predicted clock function was modeled across differing entrainment conditions, with all combinations of photoperiod length (3–21 h), daytime temperature (17, 21, 25, or 29°C), and day/night temperature differential (4, 8, or 12°C). The highest temperature regime (29°C day) resulted in clock dysfunction, although clock function was restored under SD conditions with large day/night temperature differentials of 8°C or 12°C. However, the large temperature differentials led to the loss of entrainment in SD under low temperature (17°C day; Avello et al., 2019). These results indicate that the higher and more extreme temperatures and warmer nighttime temperatures associated with climate change in the absence of corresponding changes in light conditions may disrupt temperature entrainment and, more broadly, clock function in general.

The interaction between time of day and temperature signaling can also influence clock function. In Arabidopsis grown under LD conditions, a 4-h temperature cycle of ±10°C given at different times of day causes unique effects on the oscillator (Masuda et al., 2020). A 10°C increase had a more dramatic effect on *CCA1* promoter expression that was time of day dependent compared to a 10°C drop, which had minimal effects overall. The 10°C increase was associated with greater fresh weight when given at 8 h compared to 14 h after dawn, with a linear relationship between *CCA1* amplitude and growth (Masuda et al., 2020). This response may be tuned to the expectation that the warmest part of the day usually occurs in the mid-afternoon rather than near dusk (Figure 1). A similar study imposing a temperature change from ambient 22°C to 28°C for 3 h at various times during a 12-h photoperiod resulted in differential effects on clock gene expression (Mizuno et al., 2014). Nighttime increases in temperature of 6°C had dramatic effects on clock gene expression, with a gradual increase in expression throughout the night of *PRR7*, *PRR9*, *GI*, and *LUX* that was dependent on the presence of *ELF3* and *ELF4* (Mizuno et al., 2014). While nighttime temperature increases are less common in natural settings on an acute scale, more land area has experienced asymmetric nighttime versus daytime warming globally (from 1983 to 2017; Cox et al., 2020). Warmer night temperatures (2°C–3°C increase) led to a 12.5% reduction in grain yield and altered global temporal transcriptional patterns in field-grown rice panicles, with circadian and diel rhythmic genes most sensitive to the warmer nights (Desai et al., 2021). The time of day when temperature fluctuations occur on both daily and seasonal scales is relevant when considering the impact of temperature variation and climate change on clock function and plant growth and performance.

**Figure 3:**
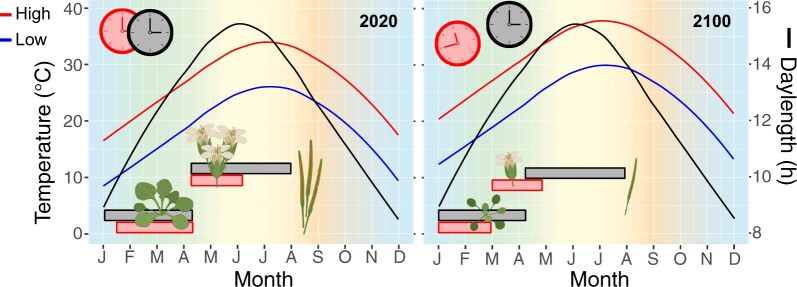
Potential thermomorphogenesis and clock misalignment with climate change. Model of current (2020) and future (2100) annual high and low temperature (°C) and daylength (h) patterns for New Orleans, LA, USA (30.0°N, 90.0°W). Bars represent the time period of clock signaling for flowering (top) and growth (bottom) based on daylength and temperature. High ambient temperatures can cause petiole elongation, hyponasty, early flowering, and reduced flower and seed number (Casal and Balasubramanian, 2019; Lippmann et al., 2019). As global temperatures rise, daylength patterns will remain unchanged; this may result in misalignment of clock signaling, premature flowering, and reduced fitness in plants adapted to current conditions. Temperature and daylength data from Weather Spark (https://weatherspark.com/); temperature data for 2100 modeled based on 4°C temperature rise with climate change (Tollefson, 2020); graphics created with BioRender (https://biorender.com/).

Temperature also directly impacts flowering time through vernalization and temperature-dependent induction or repression of flowering. Vernalization is a process through which flowering repression is released after an extended period in low temperatures. CCA1 and LHY are involved in activating expression of *VERNALIZATION INSENSITIVE 3 (VIN3)*, a critical component of the vernalization pathway, especially under mild cold conditions or early during vernalization, to accelerate flowering in Arabidopsis (Kyung et al., 2022). Elevated, but not stressful, temperature (27°C versus 23°C) accelerates flowering of Arabidopsis under SD to a similar degree as LD photoperiodic induction (Balasubramanian et al., 2006). Earlier flowering persisted across several photoperiod response mutants, indicating that high temperature induced flowering is independent of photoperiodic induction (Balasubramanian et al., 2006). As climate change causes ambient temperature rise, high temperature flowering initiation may precede photoperiodic induction, resulting in altered growth, premature transition from vegetative growth to flowering, and ultimately reduced fitness traits, such as flower number or seed yield (Figure 3). Studies of circadian behavior and plant performance across elevational clines could introduce temperature variation within a photoperiodic zone to demonstrate temperature effects on clock function and flowering in natural environments.

## Circadian integration of temperature and photoperiodic signaling

There has been substantial work focused on the interplay between light signaling, temperature, and the circadian clock, but these experiments are often confined to controlled growth conditions where one condition is fixed while the other is perturbed. Increasing temperatures with consistent daylength patterns could create misalignment between light- and temperature-entrained signaling and disrupt the timing of clock-directed physiological or developmental processes (Figure 3). Alternatively, as the change in climate drives species to more northern latitudes, how will the new photoperiod and thermocycle conditions alter growth and fitness? Over 2,000 genes (13.5% and 10.7% of cycling genes in poplar and rice, respectively) are entrained uniquely by thermocycles in poplar and rice (Filichkin et al., 2011). Additionally, 11% of cycling genes in *Brassica rapa* displayed altered temporal transcript abundance patterns between photocycling and thermocycling conditions (Greenham et al., 2020). Given these global effects on the transcriptome, the loss of or shift in thermocycling with respect to photocycles is likely to have far-reaching impacts on clock function and thus growth and productivity in natural settings.

Transcriptional and post-transcriptional dynamics orchestrated by the clock coordinate plant seasonal development, growth, metabolism, and other responses. Arabidopsis recombinant inbred line (RIL) populations entrained in field conditions throughout the growing season displayed variation in clock phase of the *COLD CIRCADIAN RHYTHM RNA BINDING 2* (*CCR2*) promoter across different months, with the extent and direction of phase change variable across populations and individual RILs (Rubin et al., 2017). Interestingly, longer circadian periods were associated with reduced rosette branch number and increased cauline branching across these RIL populations (Rubin et al., 2018). In natural populations of perennial *Boechera stricta*, early above-ground biomass accumulation was positively associated with period length across families (Salmela et al., 2016).

In a study of transcriptional patterns across seasons of perennial *A. halleri* subsp. *gemmifera* in its natural environment, associations of transcriptional profiles across 2 years showed clear seasonal patterns, with varied gene ontologies enriched across different seasons (Nagano et al., 2019). Additionally, diel oscillation of core clock gene expression, such as *CCA1*, *GI*, and *TOC1*, displayed variation in amplitude across seasons, with lowest amplitude during the winter and highest in the summer (Figure 2). This reduction in amplitude during the winter season was observed in over 80% of rhythmic genes (Nagano et al., 2019). Similar loss of rhythmicity of the circadian clock was previously observed during the winter season in chestnut (*Castanea sativa*; Ramos et al., 2005). In Arabidopsis, diel patterns of transcriptional and metabolic profiles were also dampened for plants in constant low temperature conditions (Espinoza et al., 2010). Interestingly, while transcript levels of *CCA1* and *LHY* in Arabidopsis dampened within a few days at 4°C, their protein levels remained abundant and rhythmic (Kyung et al., 2022). This suggests that protein translation and degradation may serve an important role in clock-mediated cold temperature responses.

In temperate regions, seasonal temperature lags behind changes in daylength (Box 2; Figure 1), but in natural environments it is difficult to parse these effects on transcriptional dynamics. To separate these effects, Nagano et al. (2019) grew plants in chambers with a temperature lag behind daylength to mimic the natural environment, or with temperature change in-phase or anti-phase with daylength change. For plants grown under the same temperature regime with an anti-phase daylength pattern, gene expression was more highly correlated to temperature than daylength. Additionally, plants grown in “natural” lag conditions had increased fitness compared to those in the in-phase or anti-phase conditions, indicating adaptation to the natural lag between temperature and daylength (Nagano et al., 2019). Temperature was also identified as the main driver of transcriptional and metabolic variation in Arabidopsis across diel and constant light experiments at 20°C or 4°C (Espinoza et al., 2010).

Box 2 Natural lag of temperature behind photoperiod in natural environments.

The axial tilt of the earth's rotation generates disproportionate changes in solar radiation and temperature amplitudes throughout the year across latitudinal zones (Hut et al., 2013), with smaller temperature and photoperiodic ranges near the equator and more variable ranges further away (Figure 1). An important property to consider when assessing latitudinal clines is the annual hysteresis on earth that results in temperature lagging behind photoperiod (Hut et al., 2013; Donohoe et al., 2020), such that the hottest day occurs sometime after the longest day of the year (summer solstice), and likewise, the coldest day occurs after the shortest day (winter solstice). Additionally, this lag varies across the year, as there is usually a longer delay from the summer solstice to maximum temperature, and a shorter one from the winter solstice to minimum temperature. The length and extent of asymmetry of this seasonal lag period varies across locations (Donohoe et al., 2020). This temperature lag also occurs daily, with the warmest part of the day generally occurring after midday and the coldest part of the night generally near dawn (Figure 1). The seasonal and daily light and temperature patterns in natural environments therefore conflict with the environmental conditions in most controlled environment experiments, which primarily use temporally aligned and stepwise light and temperature transitions.

Along with temperature and photoperiodic variation, plants experience developmental and maturity changes across a season. Diel patterns of both clock and seed-fill genes were observed in cowpea (*Vigna unguiculata*) leaves, pods, and seeds, but patterns varied across organs and maturity stage (Weiss et al., 2018). Interestingly, divergent patterns or loss of rhythmicity were found in controlled environments compared to the field (Weiss et al., 2018). In contrast, Nagano et al. (2019) reported an association in seasonal gene expression patterns between field and growth chamber experiments, but gene expression was more highly correlated to temperature in the controlled environment compared to outdoors. Findings regarding flowering also vary across lab and natural conditions (Song et al., 2018). While FT peaks at the end of day in Arabidopsis under lab settings, Song et al. (2018) found FT peaking in both the morning and evening in natural environments. Introduction of thermocycling and a lower R/FR ratio (from 2 to 1) into controlled conditions, to better mimic natural conditions, recapitulated the bimodal peaks of FT that were observed in the natural environment (Song et al., 2018). Despite the difficulty of separating long- and short-term dynamics in natural environments, studies in natural settings include nuance that is missed in controlled environment studies that do not include stochastic fluctuations, seasonal or daily lag, and the myriad of dynamic environmental cues that occur outdoors.

## The clock’s influence on plant adaptation across geographic scales

The degree of daily and seasonal environmental variation is influenced by geographic location and specifically latitude and elevation. Plants that can adapt to this variation and maintain fitness can expand their geographic range. Studies in diverse organisms, including Drosophila (*Drosophila melanogaster*), salmon (*Oncorhynchus tshawytscha*), Arabidopsis, and *Mimulus guttatus*, have demonstrated latitudinal clines in circadian period parameters, suggesting that the clock is contributing to local adaptation (Costa et al., 1992; Michael et al., 2003; O’Malley and Banks, 2008; Greenham et al., 2017). Recent studies in several plant species have revealed additional geographic variation in the clock, especially for the domestication of crops as human migration led to the selection of traits that facilitated continued production in different latitudinal zones (reviewed in Nakamichi, 2014; Müller et al., 2018; Li et al., 2020). The conservation of genetic variation in circadian traits in wild plant populations provides strong evidence for a role of the clock in local adaptation.

In Arabidopsis, circadian regulated leaf movement was used to uncover a significant positive correlation between circadian period and the maximal daylength at the latitude of origin for 150 accessions (Michael et al., 2003). A study of a natural population of *M. guttatus* found significant variation in circadian period across a latitudinal cline for annuals, with longer periods in more northern populations, but this trend was not found for perennials (Greenham et al., 2017). In the case of *M. guttatus*, annuals require proper timing of flowering to avoid extreme summer drought, whereas perennial populations grow in areas with wetter soils and flower much later in the summer (Hall and Willis, 2006). Similarly, a positive association was found between latitude and period across wild species of potato, but not in landrace or cultivated varieties which maintained a short period regardless of latitude (Hardigan et al., 2017). This contrasts with tomato (*Solanum lycopersicum*), a day-neutral plant originating in the Andean region of Ecuador and Peru. A leaf movement analysis of cultivated tomato, their wild ancestor, and distantly related wild species uncovered altered period and phase in modern cultivars (Müller et al., 2016). Cultivated tomato adapted to growth in higher latitudes had a circadian period around 2-h longer with a 3-h phase delay compared to ancestral *Solanum pimpinellifolium*. Quantitative trait locus mapping uncovered the *EMPFINDLICHER IM DUNKELROTEN LICHT 1* (*EID1*) locus contributing to the phase effect and *LNK2* to the period effect (Müller et al., 2016). Plants with the cultivated *EID1* allele are shorter, flower later, and have higher chlorophyll content, demonstrating adaptive variation (Müller et al., 2016, 2018). In Arabidopsis, *LNK* genes connect the circadian clock with phytochrome-dependent light signaling (Rugnone et al., 2013). In tomato, this is also true, with the *LNK2* allele phenotypes being dependent on PHYB (Müller et al., 2018). The alteration to clock regulation of light signaling may be an important adaptation to the longer summer days in Europe.

Similar geographic phase associations have been found for *GI*, where the peak expression time across five photoperiods from 8 h to 16 h was associated with geographic origin across 77 Arabidopsis accessions (de Montaigu et al., 2015). Interestingly, the peak time variation within photoperiods was greatest at 12 h and smallest at 8 h (de Montaigu et al., 2015). de Montaigu and Coupland (2017) found a significant correlation between the change of daylength at the site of origin and *GI* sensitivity to daylength, defined as the difference in peak time between 12-h and 16-h photoperiods. The variation in *GI* expression also correlated with growth rate, again indicating an adaptive role for clock variation (de Montaigu and Coupland, 2017). Common garden experiments with diverse accessions or species with varying clock parameters (such as periods or phases) or sensitivities across latitudinal and altitudinal clines could experimentally couple these trends with adaptive advantage.

Not all geographic studies uncover such clear clines in circadian parameters across wild populations. Studies using *B. stricta* collected from southeastern Wyoming found a range of circadian periods among and within populations (Salmela et al., 2016). In *Mimulus laciniatus* with an annual life habit growing in the Sierra Nevada, eight populations assessed for circadian period by leaf movement were found to vary significantly in period across populations, but their period had no clear association to geographic distribution (Leinonen et al., 2020). Common to the *B. stricta* and *M. laciniatus* experiments are sampling at high elevations of 2,500–3,000 m and 1,000–2,600 m, respectively (Salmela et al., 2016; Leinonen et al., 2020). Elevational clines impose additional environmental variation across the year, altering temperature and precipitation patterns. Hut et al. (2013) reported an average decrease of temperature of 6.5°C for every 1,000-m change in altitude. Across Arabidopsis accessions from around the world, higher altitude has been correlated with shorter periods (Edwards et al., 2005). While period shortened in response to growth in warmer temperatures, accessions had diverse temperature compensation patterns. Notably, at the cool temperature (12°C) high altitude accessions had a period ∼24 h while lowland accessions averaged ∼26 h, meanwhile at warm temperatures (22°C–27°C) the lowland accession period was ∼24 h (Edwards et al., 2005). It could be that accessions from higher altitudes have adapted their clock to the cooler ambient temperatures at higher altitudes by decelerating their clock to maintain a period of ∼24 h. Although, when the few high elevation samples were removed from the dataset, the elevational effect on period was lost (Edwards et al., 2005). Similarly, circadian period did not covary with elevation across the Sierra Nevada populations of annual *M. laciniatus* (Leinonen et al., 2020), suggesting that other pressures of selection beyond elevation contribute to variation in period lengths.

## Concluding remarks

While controlled environment studies continue to elucidate the vast influences of the clock and specific mechanisms of regulation, they cannot encompass the complex effects of the clock on plants in natural environments. Environmental conditions that influence the clock naturally vary across days, seasons, and years, both in predictable and unpredictable ways. The effects of these expected patterns and unexpected events on the circadian clock likely influence plant response and ability to survive and adapt, with far-reaching implications for conservation and agriculture. We have focused on the two strongest and most consistent environmental inputs to the circadian clock, light and temperature, and highlighted how these circadian drivers vary across spatial and temporal scales with consequent implications for plant performance in natural environments. The mounting evidence of the clock’s influence on the survival, productivity, and fitness in both natural populations and field settings should prompt investigation on the influences of the circadian clock in more natural settings, across geographic and temporal scales (see Outstanding Questions). Future studies in natural seasonal environments and common garden experiments with genotypes with diverse clock traits are needed to uncover the dynamics of clock behavior and plant processes over time and space. This will be key to the adaptation of plants in natural populations and in agricultural germplasm, with widespread implications on ecosystem level conservation and on agricultural productivity in our changing climate.

ADVANCES

- Photoreceptors and thermosensors, namely, PHYB and ELF3, play an important role in integrating light and temperature cues into the clock, which in turn modulates the response to control the timing of developmental processes.
- The clock is tuned to seasonal rhythms of light and temperature, globally influencing transcriptional patterns across the year and coordinating seasonal timing of physiological processes (germination, photosynthesis, growth, flowering, dormancy, etc.).
- Natural variation in circadian clock performance across geographic scales supports contribution to local adaptation.
- Despite the challenges of differentiating environmental signals, studies in natural settings provide important insights into circadian contributions to plant adaptation.

OUTSTANDING QUESTIONS

- Following annual rises in temperature due to climate change, how will variation in circadian temperature compensation in the context of stable photoperiodic fluctuations influence species adaptation, growth, development, and range?
- How are desynchronized thermocycle and photoperiod interpreted by the clock to mediate diverse processes?
- What are the differences in clock responses between prolonged changes (e.g. global rising temperatures) versus changes in acute cycling and weather events?
- How does clock plasticity versus persistence affect trait stability (growth, development, yield) across years, seasons, and environments?

## Notes

M.W.O. and K.G. conceived of the structure and contents of the manuscript. M.W.O. prepared the figures. M.W.O. and K.G. wrote the manuscript.

The author responsible for distribution of materials integral to the findings presented in this article in accordance with the policy described in the Instructions for Authors (https://academic.oup.com/plphys/pages/General-Instructions) is: Kathleen Greenham (greenham@umn.edu).
